# Supplementary material for: Problem-Based Learning: A Case of Acral Melanoma
Source: MedEdPORTAL. 2026 Jun 2;22:11605. doi: 10.15766/mep_2374-8265.11605 (PMC13226643; doi:10.15766/mep_2374-8265.11605)
Supplement: Supplementary file 1 — PBL Case Facilitator Guide.docxStudent Materials.docxStudent Survey.docx [file mep_2374-8265.11605-s001.zip › A. PBL Case Facilitator Guide.docx]

*Facilitator’s notes are in blue italics.*

**Day 1.**

*Ask a student to read the patient identification statement.*

**Samuel Davidson (he/him) is a 56-year-old male with a past medical history of essential hypertension, dyslipidemia, angina, type 2 diabetes mellitus, and tobacco use disorder who presents to an urgent care clinic with a wound on his left foot.**

*Provide the student group with the following information as they request it. Facilitators may vary with how much content from each section they provide depending on the group’s experience with PBL and time allotted for the day’s session.*

| **Social Determinants of Health (responses to pre-visit questionnaire)** | |
| --- | --- |
| We ask all patients the following questions because they are known to affect overall health | |
| In the past year, were there times when you didn’t have enough money to buy food? | No |
| In the past year, were you unable to pay your utility bills (such as electricity, water, or gas)? | No |
| In the past year, were you unable to pay your rent or mortgage? | No |
| In the past year, did you miss a medical appointment because you didn’t have a way to get to the clinic or hospital? | No |
| Do you currently have health insurance? | Yes |

| **History of Present Illness (OLD CAAARS)** | |
| --- | --- |
| Onset | Stepped on broken glass and cut his foot a few months ago in his garage. He did not seek care. The initial wound healed in about a week, but there now appears to be a new wound at the same location. |
| Location | Side of left foot |
| Duration | A few months |
| Characteristic | Non-healing wound |
| Associated symptoms | Mildly tender |
| Aggravating | Mildly tender when walking |
| Alleviating | Tried topical antibiotic ointment without improvement |
| Radiation | Not applicable |
| Severity | Reasonably concerned as this wound has not healed |

| **Patient Perspective** | |
| --- | --- |
| Patient’s explanatory model of illness | He is worried that his wound might be infected, and would like to determine the best plan for treatment |
| Life impact | Can be bothersome with activity, but otherwise not limiting his daily activities |
| Goal for visit | Would like to know if the wound is infected |

| **Review of Systems** | |
| --- | --- |
| Constitutional | No fevers, chills, sweating, weight changes, or fatigue |
| HEENT | No vision changes, eye pain, photophobia, hearing changes, rhinorrhea, congestion, nosebleeds, sore throat, dysphagia, or odynophagia |
| Cardiovascular | No chest pain, palpitations, orthopnea, paroxysmal nocturnal dyspnea, or edema |
| Pulmonary | No cough, hemoptysis, wheezing, or stridor |
| Gastrointestinal | No nausea, vomiting, heartburn, abdominal pain, diarrhea, blood in stools, or jaundice |
| Genitourinary | No difficulty urinating, dysuria, urgency, frequency, hematuria, incontinence |
| Lymphatic | No swollen or tender lymph nodes |
| Neurologic | No headache, dizziness, facial droop, slurred speech, tremor, numbness or tingling, weakness, ataxia, syncope, or seizures |
| Musculoskeletal | No arthralgias, myalgias, or joint swelling. **Pain at base of the left 5^th^ toe.** |
| Skin | **Wound as mentioned.** No other concerning skin lesions. |
| Endocrine/Thyroid | No polyuria or polydipsia. No hot/cold intolerance, hair changes, or neck masses |
| Psychiatric | No depression, anxiety, hallucinations, or suicidal ideation |

| **Medical History** | |
| --- | --- |
| Past medical history | Essential hypertension  Angina  Type 2 diabetes (last hemoglobin A1c was 6.5%, checked two months ago)  Dyslipidemia  Tobacco use disorder |
| Childhood Illnesses | No major illnesses or hospitalizations |
| Past surgical history | No surgeries |
| Medications | 1. Metoprolol tartrate 12.5 mg orally 2 times daily (cardio selective beta-1 receptor blocker). No recent missed doses. 2. Losartan 25mg daily (angiotensin receptor blocker). No recent missed doses 3. Nitroglycerin 0.4 mg as needed (vasodilator for angina). Does not recall last use. 4. Metformin 1000 mg twice daily (reduces hepatic gluconeogenesis, increases insulin sensitivity). No recent missed doses. 5. Glargine, injected once daily (long-acting insulin). No recent missed doses. 6. Atorvastatin 40mg once daily (HMG CoA reductase inhibitor). No recent missed doses. |
| Allergies | No known allergies |
| Family history | Mother (78): hypertension and diabetes. Father (76): heart disease. Five siblings, all relatively healthy. |
| Living situation | Lives with his wife of 30 years and cat. Enjoys card games and horror movies. |
| Diet | Relatively well-balanced diet. Would like to cut back on red meat. |
| Employment | Tire shop foreman, been at this job for 21 years. Enjoys his work. |
| Alcohol use | None |
| Tobacco use | Smokes ½-1 pack daily for 39 years |
| Illegal drug use | None |
| Sexual history | Sexually active with wife. No other partners. |
| Safety | Feels safe in his relationship. No firearms in home. |
| Travel | Traveled to Mexico 6 months ago. No other significant travel. |

| **Physical Exam** | |
| --- | --- |
| Vitals | T: 35.6 C  HR: 72 bpm  BP: 113/72 mmHg  RR: 16 bpm  SpO2: 99% RA  BMI: 27.06 kg/m2 |
| General | Well-appearing, no acute distress, not diaphoretic |
| HEENT | Pupils equal and reactive to light. Sclerae anicteric. Conjunctivae normal. Nares and tympanic membranes normal. |
| Neck | No cervical lymphadenopathy or thyromegaly |
| Cardiac | Regular rate and rhythm. Normal S1, S2. No gallops or murmurs. JVP < 3 cm. Normal carotids. 2+ dorsalis pedis and posterior tibial pulses. No pedal edema. Capillary refill < 2 seconds |
| Pulmonary | Clear to auscultation bilaterally. No wheezing or rales. |
| Abdomen | Soft, non-tender. Normal bowel sounds. No hepatosplenomegaly. |
| GU and rectal | Declined |
| MSK | Normal strength and range of motion |
| Neuro | Oriented to person, place, and time. No focal deficits. No motor or sensory deficits of the affected foot. Monofilament testing was normal. |
| Skin | **See photograph** |
| Psych | Normal mood and affect |


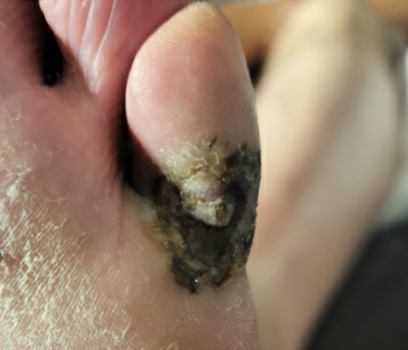

Image: Author owned

*Ask students: How would you describe this skin lesion?* *Use these prompts to guide a systematic description:*

- *Where is this lesion located?*
- *What color(s) are present?*
- *What is the primary lesion (macule, patch, papule, plaque, vesicle, bulla, nodule, tumor, pustule, wheal, cyst)?*
- *What are the secondary features (scale, crust, excoriation, fissure, erosion, ulcer, purpura, atrophy, lichenification)?*

*Example description (note: multiple are possible): Located on the plantar base and lateral aspect of the left 5^th^ toe is an ulcerated pink and dark brown plaque with overlying white/yellow and brown crust. Scale is noted on the ball of the foot.*

*Ask students to generate a problem list: answers below*

1. *Non-healing ulcer of toe*
2. *Essential hypertension*
3. *Angina*
4. *Dyslipidemia*
5. *Type 2 diabetes mellitus*
6. *Tobacco use disorder*

*Ask students to generate a differential diagnosis using VINDICATE P: potential diagnoses listed below*

- *Vascular*
  - *Peripheral artery disease*
  - *Thromboangiitis obliterans (Buerger disease)*
  - *Thrombotic disorder*
- *Inflammatory*
  - *Gout*
- *Neoplastic/Nutrition*
  - *Squamous cell carcinoma*
  - *Melanoma*
  - *Kaposi sarcoma*
- *Drugs/Toxins*
  - *Possible undisclosed salve. Note: it is not uncommon for patients to try topical products (including escharotic agents) recommended by friends/family or online sources (e.g., search “Black salve” as an example).*
- *Infectious*
  - *Osteomyelitis*
  - *Ecthyma gangrenosum (rapidly progressive ulcerative lesion from perivascular infection with pseudomonas)*
  - *Mycetoma (chronic, slowly progressive infection of the skin and subcutaneous tissue)*
  - *Endocarditis (septic emboli)*
- *Congenital*
  - *Not applicable*
- *Autoimmune*
  - *Raynaud’s phenomenon*
- *Trauma*
  - *Foot trauma*
- *Endocrine*
  - *Diabetic foot ulcer*
- *Pregnancy/Psychiatry*
  - *Not applicable*

***Ask students:***

*Are there labs or studies you would like to order?*

*What are you looking for?*

The urgent care clinic obtained the following:

- Labs: CBC with differential, ESR, CRP
- Imaging: X-ray of the foot
- *Note: Students may wish to request additional labs (e.g., CMP). Ask them to explain what additional data they would seek and why.*

| **Complete Blood Count with Differential** | | **Reference Range** |
| --- | --- | --- |
| Hemoglobin | 14.6 | 13.5-17.5 g/dL |
| Hematocrit | 44.8 | 41-53% (male) |
| Mean corpuscular hemoglobin (MCH) | 27.7 | 25-35 pg/cell |
| Mean corpuscular volume (MCV) | 84.8 | 80-100 um^3^ |
| Mean corpuscular Hgb conc (MCHC) | 32.6 | 31-36% Hb/cell |
| White blood cells | 7.78 | 4.5-11.0 k cells/mm^3^ |
| Neutrophils | 61.7 | 54-62% |
| Neutrophils, bands | 3.5 | 3-5% |
| Lymphocytes | 23.4 | 25-33% |
| Monocytes | 9.0 | 3-7% |
| Eosinophils | 1.8 | 1-3% |
| Basophils | 0.6 | 0-0.75% |
| Platelets | 302 | 150-400 k cells/mm^3^ |

| **Inflammatory markers** | | **Reference range** |
| --- | --- | --- |
| CRP | **2.8** | 0.0-0.8 mg/dL |
| ESR | **18** | 0-15 mm/h |


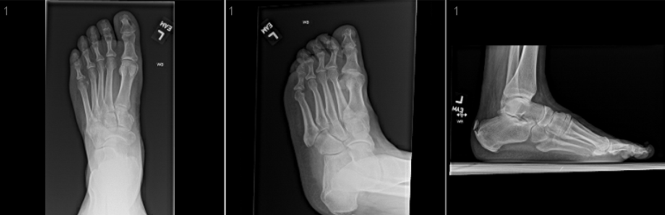


Image: Author owned

X-ray impression: No osseous erosion or destruction to suggest acute osteomyelitis. Consider evaluation with MRI (with and without contrast) if there is continued concern for osteomyelitis.

*Ask students what they would recommend next.*

- *Any additional labs or imaging?*
- *Would you consult any specialists?*
- *What treatment steps would you take at this point?*

A referral was placed to orthopedics. The orthopedic service saw Mr. Davidson within a few days and recommended an MRI.


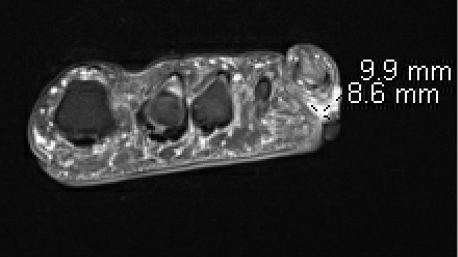

Image: Author owned

MRI impression: Ulceration at the lateral/plantar aspect of the fifth proximal interphalangeal joint with small adjacent superficial fluid collection. Associated cellulitis. No MR findings (T1 hypointense enhancing marrow edema) of acute osteomyelitis.

The orthopedic team recommended a referral to dermatology for further evaluation of the skin lesion. Their referral stated, “56-year-old male with previous history of foot trauma, now with non-healing wound and no evidence of osteomyelitis – concern for neoplasm.”

*This is the end of Day One.*

*Ask students to finalize their learning issues. Note: students often generate potential learning issues throughout the session. Now is the time to finalize them.*

*Potential learning issues:*

- *What are the basic principles of wound healing (e.g., stages, cells types, and signaling molecules involved)?*
- *What is the differential diagnosis for a foot ulcer?*
- *What are the clinical features of osteomyelitis?*
- *What radiographic findings are consistent with osteomyelitis?*
- *What skin cancers typically present on the foot?*

**END OF DAY ONE**

**DAY 2.** *Start this session with students teaching from their learning products generated on Day 1. Time per learning product depends on the time available for this session.*

**Continuation of case:** Mr. Davidson was evaluated in dermatology clinic. This clinical photograph was taken at that time.

*Ask students to describe the findings.*


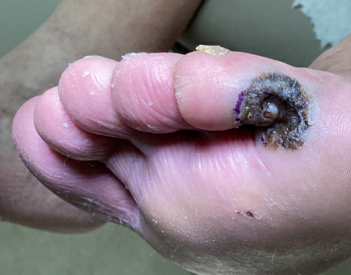


Image: Author owned

*Provide the following information:* The dermatologist noted a dark brown-black ulcerated plaque with maceration at the periphery on the lateral and plantar surface of the 5^th^ toe. A total body skin exam was performed and notable for a few actinic keratoses on the scalp, face, and forearms, as well as scattered melanocytic nevi on the trunk and extremities *(VisualDx would be an excellent resource if students would like to reference examples of actinic keratoses and nevi)*. The dermatologist was concerned the lesion on the foot could be a melanoma and performed a skin biopsy (the site was marked with a purple surgical marker). Results are shown here *(students are not expected to describe this histology – no need to ask them to interpret the findings)*:


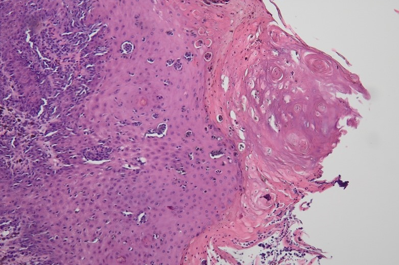

Image: Author owned

The biopsy revealed a T3b acral malignant melanoma with a Breslow thickness of at least 3.2 mm. *Students may want to look up the definition of Breslow depth at this point.*

Mr. Davidson was informed of the diagnosis by telephone within 48 hours of the biopsy results. After the procedure, he became increasingly worried and searched online for information about melanoma. However, he found much of the information confusing and difficult to interpret. This left him with many unanswered questions, including:

- How did this happen?
- Does he have a higher risk for melanoma?
- Could this be related to frequent sun exposure, even though the lesion is on his foot?

He brings up the musician Bob Marley, who died from a melanoma on the foot, and shares he fears a similar outcome. He is curious about his potential treatment options. His reliance on internet sources has heightened his anxiety. He arrives at the visit with many questions about his diagnosis and is especially curious about his treatment options.

*If time allows, consider pausing here to ask students how they might approach Mr. Davidson’s concerns, particularly any health literacy challenges and his anxiety about the diagnosis. How would they ensure he understands his condition and feels supported in making treatment decisions?*

The dermatologist acknowledged that a cancer diagnosis, especially one delivered by phone, can feel overwhelming. They reassured Mr. Davidson that it was completely understandable for him to search for more information and to feel anxious about what he found. They noted that while the internet can offer useful content, it often includes conflicting or overly technical information that can be confusing and frightening. They took time to explain what melanoma is, how it develops, and what it meant in his specific case. They responded empathetically to his reference to Bob Marley, acknowledging the fear this association brought up, and emphasized that every case is unique and treatable in different ways. To help him better understand his diagnosis and options, the dermatologist provided written materials in plain language and directed him to trusted patient resources. They paused frequently to check his understanding and invited him to ask additional questions. They offered to connect him with a patient navigator who could support him through the next steps.

An expedited referral was placed to a multidisciplinary team with expertise in melanoma treatment. He was informed that:

- Additional testing (e.g., cancer genetics) would be performed on the biopsy specimen to guide treatment, including the potential for immunotherapy.
- He would likely undergo a PET CT to assess the extent of disease.
- He would meet with both surgical and medical teams to discuss treatment options, including systemic therapy.

*This is the end of Day Two.*

*Ask students to finalize their learning issues.*

*Potential Learning Issues:*

- *What is the morphology of melanoma? What features distinguish between the major subtypes?*
- *Does melanoma present differently on different skin types? What* *is the Fitzpatrick phototype scale?*
- *Why is melanoma considered a health care disparity? What racial disparities exist in melanoma detection and outcomes?*
- *Who is at risk for developing melanoma? What genetic mutations are commonly associated with familial and sporadic melanomas?*
- *What patient education should be provided for skin cancer prevention? What are the ABCDEs of melanoma?*
- *What is the relationship between UV radiation and melanoma pathogenesis? What DNA repair mechanisms are most relevant for correcting UV-induced damage?*
- *What is Breslow Depth, and how does it relate to prognosis? How is a melanoma staged?*
- *What are the general principles of melanoma treatment? How do immune checkpoint inhibitors work, and why are they effective in highly mutated cancers? How do BRAF inhibitors work?*
- *How does a PET scan work, and how are the results used in prognosis?*

**END OF DAY 2.**

**DAY 3.** Start with students teaching from their learning products generated on Day 2. Time per learning product depends on the time available for this session.

*Ask a student to provide a summary of the case.*

*Example summary:* Samuel Davidson is a 56-year-old male with a past medical history of essential hypertension, angina, dyslipidemia, type 2 diabetes mellitus, and tobacco use disorder who presented to an urgent care clinic with a non-healing wound on his left foot. Examination revealed a necrotic ulcer on the left 5^th^ toe, initially concerning for osteomyelitis. However, imaging did not support this diagnosis. He was later evaluated by dermatology, where a skin biopsy revealed an acral melanoma. He was referred to a local cancer center with expertise in melanoma.

*Provide the following information:*

**Problem List**

1. New diagnosis of acral melanoma
   - A PET-CT demonstrated metastatic melanoma, with several skin lesions and lymph nodes involved, extending from the fifth digit of the left foot to the level of the mid-thigh.
   - Mr. Davidson met with the surgical oncology team. He underwent toe amputation with excision of in-transit metastasis *(cancer cells spreading from the primary tumor along the lymphatic vessels)* on the dorsal foot.
   - Tumor genetic analysis revealed: BRAF wild type, NRAS mutation, and copy number gain in CRKL, MAPK1. Given this molecular profile, the decision was made to initiate monotherapy with an anti-PD1 agent (pembrolizumab).


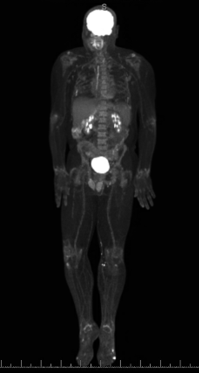


Image: Author owned

*Ask students to work through the Mechanism Of Disease or Concept Map at this point. We recommend saving the clinical outcome below until students have worked through their conceptual models.*

*Provide the following information:*

Unfortunately, Mr. Davidson’s disease did not respond to immunotherapy. He developed progressive peritoneal disease, malignant ascites, and died from his cancer less than 18 months after his initial diagnosis.

END OF DAY 3.

*Students will be provided with the following list of learning objectives at the end of the last session.*

**Learning Objectives:**

- List the typical stages of wound healing and describe the common cell types and signaling pathways involved
- Generate a differential diagnosis for a non-healing foot ulcer
- Describe the clinical and radiographic features that support a diagnosis of osteomyelitis
- Identify and describe the morphology of melanoma in all skin types, including characteristic features of the major types (superficial spreading melanoma, nodular melanoma, lentigo maligna melanoma, acral lentiginous melanoma)
- List the most significant risk factors for developing melanoma, including genetic, environmental, and phenotypic contributors
- Explain the ABCDE mnemonic for melanoma detection and describe its use in patient education for skin self-examinations
- Describe the key components of patient education for skin cancer prevention, including sun protection strategies, risk factor awareness, and routine skin examinations
- Explain how defects in DNA repair pathways, dysregulated signal transduction, and abnormal cell cycle control contribute to melanoma pathogenesis
- List the most common genetic mutations in melanoma and explain their role in tumor development and progression
- Explain how Breslow depth is measured and its prognostic significance in melanoma
- Describe the basic principles of melanoma treatment, including indications for surgery, immunotherapy, and targeted systemic therapy
- Explain how immunotherapies work and describe why this approach is effective with cancers like melanoma that display high mutation rates
- Discuss melanoma as a healthcare disparity (e.g., differences in detection, diagnosis, outcomes, and access to care across diverse populations)

**Session Objectives**:

1. Identify cutaneous morphology concerning for melanoma
2. Provide patient education around melanoma risk
3. Discuss melanoma as a health care disparity
