# Supplementary material for: Problem-Based Learning: A Case of Acral Melanoma
Source: MedEdPORTAL. 2026 Jun 2;22:11605. doi: 10.15766/mep_2374-8265.11605 (PMC13226643; doi:10.15766/mep_2374-8265.11605)
Supplement: Supplementary file 1 — PBL Case Facilitator Guide.docxStudent Materials.docxStudent Survey.docx [file mep_2374-8265.11605-s001.zip › C. Student Survey.docx]

**Melanoma PBL Survey**

Last edited April 30, 2025

*Note: This survey was developed for the purposes of this study and is not administered routinely with every PBL session. It is recommended when more granular feedback about the case is desired.*

Prior to completing the melanoma case, how confident or unconfident were you in recognizing melanoma skin cancer?

- Very confident
- Somewhat confident
- Neither confident nor unconfident
- Somewhat unconfident
- Very unconfident

After completing the case, how confident or unconfident do you feel in recognizing melanoma skin cancer?

- Very confident
- Somewhat confident
- Neither confident nor unconfident
- Somewhat unconfident
- Very unconfident

Prior to completing the case, how confident or unconfident were you in recognizing melanoma in all skin types?

- Very confident
- Somewhat confident
- Neither confident nor unconfident
- Somewhat unconfident
- Very unconfident

After completing the case, how confident or unconfident do you feel in recognizing melanoma in all skin types?

- Very confident
- Somewhat confident
- Neither confident nor unconfident
- Somewhat unconfident
- Very unconfident

Prior to completing the case, how knowledgeable were you of melanoma risk factors?

- Very knowledgeable
- Somewhat knowledgeable
- Slightly knowledgeable
- Not at all knowledgeable

After completing the case, how knowledgeable are you of melanoma risk factors?

- Very knowledgeable
- Somewhat knowledgeable
- Slightly knowledgeable
- Not at all knowledgeable

Prior to completing the case, how confident or unconfident were you in providing patient education about melanoma prevention?

- Very confident
- Somewhat confident
- Neither confident nor unconfident
- Somewhat unconfident
- Very unconfident

After completing the case, how confident or unconfident are you in providing patient education about melanoma prevention?

- Very confident
- Somewhat confident
- Neither confident nor unconfident
- Somewhat unconfident
- Very unconfident

How well did this case lend itself to addressing a health care disparity in dermatology, if at all?

- Very well
- Somewhat well
- Not at all

Overall, how did this case compare to other PBL cases you have worked through in the curriculum? Was it…

- Much better
- Somewhat better
- About the same
- Somewhat worse
- Much worse

[if somewhat or much better selected]: In what ways was the case better than most other PBL cases?

- Open ended

[if somewhat or much worse]: In what ways was the case worse than most other PBL cases?

- Open ended

How applicable was the case to your current level of medical education?

- Very applicable
- Somewhat applicable
- Slightly applicable
- Not applicable at all

[skip this if they select somewhat applicable or very applicable] Please provide any suggestions on how the applicability of the case could be improved: [Open-ended response]

How would you rate the level of complexity of the case for your level of education and training?

- It was too advanced
- It was appropriate
- It was too basic

To what extent did the case require you to use your critical thinking and problem-solving skills, if at all?

- Very much
- Somewhat
- Not much
- Not at all

How useful were the learning products created by your peers?

- Very useful
- Somewhat useful
- Slightly useful
- Not useful at all

What aspects of the peer-generated learning projects were particularly effective or ineffective? [Open-ended response]

The following is the list of learning objectives for this case. For each objective, please indicate whether it was sufficiently address with Yes or No.

- List the typical stages of wound healing and describe the common cell types and signaling pathways involved
- Generate a differential diagnosis for a non-healing foot ulcer
- Describe the clinical and radiographic features that support a diagnosis of osteomyelitis
- Identify and describe the morphology of melanoma in all skin types, including characteristic features of the major types (superficial spreading melanoma, nodular melanoma, lentigo maligna melanoma, acral lentiginous melanoma)
- List the most significant risk factors for developing melanoma, including genetic, environmental, and phenotypic contributors
- Explain the ABCDE mnemonic for melanoma detection and describe its use in patient education for skin self-examinations
- Describe the key components of patient education for skin cancer prevention, including sun protection strategies, risk factor awareness, and routine skin examinations
- Explain how defects in DNA repair pathways, dysregulated signal transduction, and abnormal cell cycle control contribute to melanoma pathogenesis
- List the most common genetic mutations in melanoma and explain their role in tumor development and progression
- Explain how Breslow depth is measured and its prognostic significance in melanoma
- Describe the basic principles of melanoma treatment, including indications for surgery, immunotherapy, and targeted systemic therapy
- Explain how immunotherapies work and describe why this approach is effective with cancers like melanoma that display high mutation rates
- Discuss melanoma as a healthcare disparity (e.g., differences in detection, diagnosis, outcomes, and access to care across diverse populations)

[if any responses selected to the prior question] In what ways were these learning objectives unclear or insufficiently addressed? [Open-ended response]

What aspects of the PBL case were most beneficial to your learning? [Open-ended response]

What improvements would you suggest for future PBL cases? [Open-ended response]

Demographics

Finally, just a few more questions. This information is confidential and will not be reported in connection with your responses.

To which gender do you identify?

- Female
- Male
- Non-binary
- Transgender female
- Transgender male
- Other, please specify: ____________
- Prefer not to answer

Which race do you identify as? (Select all that apply)

- African American or Black
- American Indian or Alaska Native
- Asian
- Hispanic or Latino
- Native Hawaiian or Pacific Islander
- White
- Other
- Prefer not to answer

Thank you for your help in evaluating this case!
