# Supplementary material for: Problem-Based Learning: A Case of Acral Melanoma
Source: MedEdPORTAL. 2026 Jun 2;22:11605. doi: 10.15766/mep_2374-8265.11605 (PMC13226643; doi:10.15766/mep_2374-8265.11605)
Supplement: Supplementary file 1 — PBL Case Facilitator Guide.docxStudent Materials.docxStudent Survey.docx [file mep_2374-8265.11605-s001.zip › _Educational Summary Report_11605.pdf]

MedEdPORTAL
THE AAMC JOURNAL OF TEACHING AND LEARNING RESOURCES

Original Publication

Open Access

Problem-Based Learning: A Case of Acral Melanoma

Annabelle Huntsman[†], Amanda Jiang[†], Justin Lyon, Rachel R. Codden, MPH, Morgan M. Millar, PhD, Dekker C. Deacon, MD, PhD, Sarah D. Cipriano, MD, MPH, MS*

*Corresponding author: Sarah.Cipriano@hsc.utah.edu

[†]Co-primary author

Abstract

Introduction: Dermatology-focused problem-based learning cases are underrepresented in medical education despite the importance of skin cancer recognition across specialties. This project aimed to design, implement, and evaluate a melanoma-focused problem-based learning case for first-year medical students. **Methods:** We developed a problem-based learning case on acral melanoma with input from dermatologists, medical students, and problem-based learning course directors. The case was piloted, revised, and incorporated into the curriculum at the Spencer Fox Eccles School of Medicine. Evaluation included a postcase anonymous survey to assess changes in confidence via a retrospective pre/post survey. **Results:** Sixty-two students completed the survey. Confidence in recognizing melanoma rose from 16.4% to 86.9% ($P < .001$). Confidence in recognizing melanoma in all skin types increased from 3.3% to 65.6% ($P < .001$). Self-reported knowledge of melanoma risk factors and confidence in prevention counseling improved from 5.1% to 55.9% and from 30.5% to 86.4%, respectively (both $P < .001$). Most students (93.2%) felt that the case addressed health care disparities. Every respondent (100%) rated the complexity of the case as appropriate for their level of education and training, and 81.3% rated it superior to other problem-based learning cases in their curriculum. **Discussion:** This melanoma problem-based learning case improved self-reported learner confidence and knowledge while addressing health care disparities in dermatology. It is adaptable across institutions and highlights the importance of including dermatology cases early in training.

Keywords
Acral Melanoma, Problem-Based Learning, Skin Cancer, Clinical Reasoning/Diagnostic Reasoning, Social Determinants of Health

Educational Objectives

By the end of this activity, learners will be able to:

1. Identify cutaneous morphology concerning for melanoma.
2. Provide patient education around melanoma risk.
3. Discuss melanoma as a health care disparity.

Introduction

Problem-based learning (PBL) is a widely used small-group, student-centered teaching modality in medical education that emphasizes self-directed learning, critical thinking, and clinical reasoning.[1-2] Evidence from systematic and scoping reviews suggests that PBL is at least as effective as traditional lecture-based teaching for knowledge acquisition, and often superior for developing problem-solving, self-learning, communication, and clinical skills.[1-2] PBL also tends to achieve high levels of student satisfaction and better prepares learners for real-world clinical practice.[1]

Although *MedEdPORTAL* offers a wide range of published PBL cases spanning multiple clinical disciplines, to our knowledge there are no cases dedicated to skin cancer detection, prevention, and management.[3-7] A significant educational gap exists given that skin diseases are prevalent: 1 in 4 patients of all ages in the United States annually seek care for a skin condition.[8] Melanoma education is particularly critical, as prognosis depends heavily on early detection, with survival rates decreasing sharply as melanoma thickness increases.[9] Because of limited dermatology access, it is essential to train all physicians, not just dermatologists, in basic skin cancer examinations. Clinician-detected melanomas, whether through directed skin exams or opportunistic screening during routine physicals, are thinner than those found by patients or their family members.[9] Therefore, all physicians must be able to recognize suspicious lesions, counsel patients on risk factors, and facilitate timely referrals.

Citation:
Huntsman A, Jiang A, Lyon J, et al. Problem-based learning: a case of acral melanoma. *MedEdPORTAL*. 2026;22:11605.
https://doi.org/10.15766/mep_2374-8265.11605

Copyright © 2026 Huntsman et al. This is an open-access publication distributed under the terms of the Creative Commons Attribution license.

In addition, disparities in skin cancer outcomes highlight the need to integrate equity considerations into dermatology education.[10] Acral melanoma, for example, disproportionately affects patients with darker skin tones, yet it is often diagnosed at later stages due to underrecognition. Teaching students to recognize these variations early in their training can improve both diagnostic accuracy and awareness of health inequities.

Our PBL case was designed to introduce first-year medical students to the complexities of melanoma diagnosis through a real-patient case unique to physicians across multiple specialties. The objectives were to improve students' confidence in melanoma detection and management, highlight interdisciplinary care, and address health equity issues in dermatology. We also evaluated the case's effectiveness and student satisfaction in meeting these educational goals.

Several educational innovations have sought to improve dermatology training for undergraduate medical students. Reviews of dermatology teaching methods have identified a broad range of approaches, including various forms of e-learning (gamification, asynchronous modules), observed clinical exams with standardized patients and moulage, podcasting, and interdisciplinary clinical electives.[11] Our curriculum contributes to this literature in 4 distinct ways: (1) embedding melanoma education within a multisession PBL framework early in medical school; (2) utilizing a less common melanoma subtype occurring in a body location frequently overlooked during routine skin exams; (3) incorporating learning objectives addressing morphologic variation across skin tones and principles of health equity; and (4) formally evaluating changes in learner confidence, perceived preparedness, and attainment of the case learning objectives. By integrating dermatologic content, clinical reasoning, health equity, and structured assessment within a replicable PBL format, this case addresses a gap not fully met by existing undergraduate dermatology education strategies.

Methods

Activity Design and Development

We designed a 3-day PBL case featuring a patient with an acral melanoma, incorporating clinical presentation, diagnostic imaging, histopathology, and social determinants of health (Appendices A and B). Two academic dermatologists and 3 medical students collaborated on case development based on a real patient scenario. We intentionally selected a case presenting with an ulcer to broaden the differential and reduce anchoring bias toward melanoma. The inclusion of preceding trauma was also deliberate, as a history of trauma at the site of acral melanoma is common. The case was piloted with our first-year medical student class in the spring of academic year 2023-24. We solicited case feedback from a focus group of 10 medical students within 2 weeks of the PBL session. The case was then revised and integrated into the curriculum for the 2024-25 academic year.

Curricular Context and Learner Preparation

Our institution incorporated PBL as 1 modality within a broader, integrated curriculum. During the week this PBL was implemented, students also participated in student-led clinics, attended lectures on additional dermatologic conditions, engaged in a team-based learning session, completed asynchronous learning modules, and participated in laboratory and doctoring course sessions. Melanoma content was delivered exclusively through PBL. Assessment was similarly distributed: each weekly quiz contained 3 PBL-specific questions out of 20, and the end-of-block final exam included 2 questions derived from this PBL out of 100 questions spanning 10 weeks of curricular content.

The case unfolded across three 120-minute sessions held on Monday, Wednesday, and Friday. During each session, students gathered patient history, examination findings, and laboratory and imaging data, then constructed a mechanism-of-disease (MOD) map on the final day. The second and third sessions opened with student-led teaching based on learning products generated from the learning issues identified during case discussion. Learning products may take various forms (eg, slides, handouts, quizzes) but must address the assigned learning objective, demonstrate adequate engagement with the learning issue, and cite at least 3 expert-reviewed resources; all images must include a citation. Each learning product presentation was allotted 4-5 minutes. At our institution, PBL groups comprised 8-10 students guided by a faculty facilitator with a background in clinical care, biological sciences, and/or medical education. Facilitators received case materials (see Appendix A) 2 weeks in advance and had the opportunity to raise questions at a weekly meeting with the content creators and/or PBL curriculum directors.

Implementation

Day 1:

- *Student preparation*: Students had no prior exposure to the case materials before the initial presentation and were free to consult online academic resources as new topics emerged throughout each session. See Appendix B for the student materials.
- *Case presentation*: Each session opened with a brief patient introduction, including the patient's name, age, chief

Copyright © 2026 Huntsman et al. This is an open-access publication distributed under the terms of the Creative Commons Attribution license.

concern, and clinical setting (eg, emergency department, primary care office, urgent care). The facilitator guide (see Appendix A) structured case pacing and the sequential reveal of information as students requested or earned it, consistent with a PBL format.

- *Patient interview*: Students conducted a patient interview, with the facilitator either acting as a surrogate patient or presenting information via handouts or slides. The facilitator guided students through each component of the patient's history: History of Present Illness, Past Medical History, Past Surgical History, Medications, Allergies, Family History, Social History, and Review of Systems.
- *Physical examination and initial findings*: Students then received the patient's physical examination findings, including vital signs, a comprehensive organ systems review, and initial laboratory and imaging results. Drawing on this information, students were prompted by their facilitator to construct a prioritized problem list and differential diagnosis using the VINDICATE P framework, a metamemory technique that produces differentials by pathophysiology.[12] Students had prior exposure to this framework through their doctoring course, reinforcing consistency across curricular contexts.
- *Learning product assignment*: In the final 10 minutes of the session, students identified 8 knowledge gaps (learning issues) relevant to the case, which they researched and presented in a brief 5-minute teaching segment (learning products) at the following session. When students struggled to identify these gaps independently, facilitators offered guidance on topics of particular importance based on the case's learning objectives.

Day 2: The session opened with students teaching from their learning products, typically 5 minutes per student, before case discussion resumed. Following these presentations, students received additional clinical information, including follow-up findings, key prognostic factors, and updated laboratory and imaging results, to stimulate discussion around the ongoing management and care of the patient. In the remaining session time, students assigned learning product topics for their second and final presentation of the week.

Day 3: The final session opened with each student presenting their 5-minute learning product, followed by a resumed case discussion. Upon completion, a finalized diagnosis was established, then students were asked to compose a brief case summary. Working collaboratively, the group then constructed a MOD map using a whiteboard incorporating relevant risk factors, social determinants of health, and contributing pathophysiology. Each PBL group submitted a MOD map to the learning management system. Case learning objectives were released to students that evening to address any remaining educational gaps. The following week opened with a 10-minute review of these objectives, giving students an opportunity to raise questions or concerns about the case content and delivery.

Assessment and Evaluation

Following case completion, we assessed students' confidence in melanoma recognition, content knowledge, and perceptions of the case using an anonymous survey developed jointly with representatives from the TRIAD/Survey Design and Measurement Core at the University of Utah. The survey included both closed- and open-text questions regarding strengths, areas for improvement, and overall applicability to the medical school curriculum. The survey was created and disseminated using REDCap.[13] Closed-ended responses were summarized descriptively using counts and percentages. Chi-square tests were used to assess differences in confidence between the pre- and post-timepoints. Statistical analyses were conducted using SAS. Open-ended questions were categorized based on common "themes" that were independently coded by 2 members of our team. Any discrepancies were resolved via group discussion. A complete version of the finalized survey is provided in Appendix C. This study was approved by the University of Utah Institutional Review Board and deemed exempt from full review.

Results

Of 133 first-year medical students, 62 (46.6%) completed the survey. Respondents were 64.4% female, 28.8% male, and 6.8% all other genders or preferred not to answer. Most identified as White (72.9%), with 20.3% identifying as other races and 6.8% preferring not to report their race.

Overall, students reported substantial improvements in confidence and knowledge following the case, as shown in Table 1. Confidence in recognizing melanoma improved markedly (from 16.4% to 86.9%), as did recognition across all skin types (from 3.3% to 65.6%). Knowledge of melanoma risk factors and confidence in prevention counseling improved substantially, from 5.1% to 55.9% and from 30.5% to 86.4%, respectively. All changes in reported confidence from precase to postcase were statistically significant.

Ninety-three percent of students agreed that the case addressed disparities in dermatology, and 81% rated it more effective than other PBLs (see Table 2). Most students (86.2%) reported that this case applied to their current level of education, and every

Copyright © 2026 Huntsman et al. This is an open-access publication distributed under the terms of the Creative Commons Attribution license.

Table 1. Student Self-Reported Pre- and Post-Melanoma PBL Case Confidence and Knowledge (*N* = 62)

Question	Precase Response *n* (%)	Postcase Response *n* (%)	*P*[a]
**Confidence recognizing melanoma skin cancer**			<.001
Confident	10 (16.4)	53 (86.9)	
Neither confident nor unconfident	11 (18.0)	7 (11.5)	
Unconfident	40 (65.6)	1 (1.6)	
**Confidence recognizing melanoma in all skin types**			<.001
Confident	2 (3.3)	40 (65.6)	
Neither confident nor unconfident	10 (16.4)	14 (23.0)	
Unconfident	49 (80.3)	7 (11.5)	
**Confidence providing patient education about melanoma prevention**			<.001
Confident	18 (30.5)	51 (86.4)	
Neither confident nor unconfident	13 (22.0)	7 (11.9)	
Unconfident	28 (47.5)	1 (1.7)	
**Knowledge of melanoma risk factors**			<.001
Very knowledgeable	3 (5.1)	33 (55.9)	
Somewhat knowledgeable	26 (44.1)	25 (42.4)	
Slightly knowledgeable	22 (37.3)	1 (1.7)	
Not at all knowledgeable	8 (13.6)	0 (0.0)	

Abbreviation: PBL, problem-based learning.
[a]*P* values were calculated with chi-square tests.

respondent (100%) rated the case's complexity as appropriate for their level of education and training. Additionally, the majority of students (95%) reported that the melanoma PBL case required them to use critical thinking and problem-solving skills.

Thematic analysis of open-ended survey responses revealed several recurring themes reflecting what students felt went particularly well. First, students appreciated the clinical relevance and realism of the case, noting that it "felt like an accurate representation of what we would see in our outpatient clinics" and "I loved how the patient had to move through urgent care then ortho referral and then finally to derm, it felt very realistic." Second, students valued the complexity and educational depth, as the case required them to broaden their differential: "I liked the plot twist—it had me thinking of a wide differential but was humbling in the sense that it was something I really hadn't considered." Third, students commented favorably on the overall structure, organization, and clarity, appreciating that it was "well organized and thought out" and that "there weren't a million findings or irrelevant findings that we could chase and waste our time." Fourth, the integration across specialties was highlighted, with students valuing the opportunity to "act in multiple roles (urgent care, derm, oncology)" and practice referring across disciplines. Finally, students responded positively to the inclusion of health care disparities, noting that the case "did an awesome job tying in skin of color considerations without forcing it" and "I appreciated how the case did not fit the textbook definition of and epidemiology of melanoma. Which ultimately led us to discussing the disparities between demographics which is always an important topic."

To improve the activity, we solicited feedback by asking, "What improvements would you suggest for future PBL cases?" and received 32 responses. Six students expressed satisfaction or suggested no changes, whereas the remainder highlighted 3 main areas for improvement: stronger histology and imaging support with more description or annotation, greater inclusion of skin of color, and changes to the overall PBL structure with obtaining the learning objectives earlier in the week.

Using a threshold of 80% agreement, students rated 10 of the 13 case learning objectives as adequately covered. The lowest-rated objective was "List the typical stages of wound healing and describe the common cell types and signaling pathways involved," with 37.3% of students indicating that it was inadequately covered. In contrast, the highest-rated objectives included "Explain how Breslow depth is measured and its prognostic significance in melanoma," "Explain the ABCDE mnemonic for melanoma detection and describe its use in patient education for skin self-examinations," and "List the most significant risk factors for developing melanoma, including genetic, environmental, and phenotypic contributors," which were rated at 96.5%, 94.8%, and 91.4%, respectively.

Open-ended feedback regarding unclear or insufficiently addressed learning objectives revealed 2 main issues: the need for more narrowly defined prescriptive objectives and concerns about variability among facilitators. Some objectives were described as overly broad, making them difficult to approach effectively within the case format. Additionally, differences in

Copyright © 2026 Huntsman et al. This is an open-access publication distributed under the terms of the Creative Commons Attribution license.

Table 2. Student Perceptions About the Melanoma PBL Case (*N* = 62)

Survey Question	*n* (%)
**How well did the case lend itself to addressing a health care disparity in dermatology, if at all?**	
Very well	27 (45.8)
Somewhat well	28 (47.5)
Not at all	4 (6.8)
**How did the case compare to other PBL cases in the curriculum?**	
Much better	15 (25.4)
Somewhat better	33 (55.9)
About the same	11 (18.6)
Somewhat worse	0 (0.0)
Much worse	0 (0.0)
**How applicable was the case to your current level of medical education?**	
Very applicable	50 (86.2)
Somewhat applicable	8 (13.8)
Slightly applicable	0 (0.0)
Not applicable at all	0 (0.0)
**How would you rate the level of complexity of the case for your level of education and training?**	
It was too advanced	0 (0.0)
It was appropriate	59 (100.0)
It was too basic	0 (0.0)
**To what extent did the case require you to use your critical thinking and problem-solving skills, if at all?**	
Very much	28 (47.5)
Somewhat	28 (47.5)
Not much	3 (5.1)
Not at all	0 (0.0)
**How useful were the learning products created by your peers?**	
Very useful	21 (36.2)
Somewhat useful	26 (44.8)
Slightly useful	8 (13.8)
Not useful at all	3 (5.2)

Abbreviation: PBL, problem-based learning.

facilitator style led to inconsistent experiences across groups. Some facilitators provided transparency and active guidance to help students meet the objectives, whereas others prioritized alternative learning points, resulting in uneven coverage and a lack of standardization.

The block course director selected 5 assessment questions addressing melanoma morphology, health care disparities, and molecular content. On the quiz administered immediately following the PBL, students performed well on morphology identification (identify and describe the morphology of melanoma across all skin types, including characteristic features of the major subtypes: 94% correct) and melanoma as a health care disparity (discuss melanoma as a health care disparity: 84% correct), with comparatively lower performance on molecular content (list the most common genetic mutations in melanoma and explain their roles in tumor development and progression: 67% correct). On the final exam, administered approximately 4 weeks later, students demonstrated retained knowledge of melanoma morphology (93% correct) and Breslow depth measurement and prognostic significance (87% correct).

Discussion

This melanoma-focused PBL case substantially improved student self-reported confidence and knowledge in melanoma detection, risk factors, and patient education. It also integrated health equity considerations and emphasized interdisciplinary care. Students found the case clinically realistic and relevant, noting that the complexity was appropriate for their level of training, and that it compared favorably with other PBL cases in the curriculum. Student feedback indicated that most learning objectives were successfully achieved, with the highest-rated topics being the ABCDE mnemonic for melanoma detection, melanoma risk factors, and melanoma depth as a prognostic measure, confirming the case's effectiveness in imparting foundational knowledge crucial to future practice.

Areas for improvement were also identified. Students highlighted the need for enhanced histopathology and imaging support, as well as more consistent emphasis on objectives across facilitators. At our institution, learning objectives are intentionally withheld until the final session to encourage student-driven inquiry; however, some facilitators opted to release them earlier, creating variability in the learning experience. Future directions include adding explanatory content for histology and imaging, expanding facilitator training, and continuing efforts to integrate diverse dermatologic cases earlier in the curriculum.

Although using an actual patient case adds relevance and realism, we acknowledge that the patient's Fitzpatrick skin type is a limitation, as acral melanoma disproportionately affects patients with darker skin tones. We explicitly addressed this in our learning objectives, emphasizing the importance of recognizing melanoma across all skin tones and framing it as a health equity issue warranting both clinical awareness and broader societal attention. Despite this limitation, our results suggest that selecting a melanoma subtype with known racial and ethnic disparities in incidence, combined with a dedicated learning objective on morphologic recognition across skin tones, was associated with meaningful gains in student confidence: the proportion of students reporting confidence in recognizing melanoma across all skin tones increased from 3.3% to 65.6%.

A limitation of our assessment is that our survey relied on self-reported confidence rather than objective measures of knowledge acquisition. Although capturing changes in knowledge or behavior would yield more concrete outcomes, self-reported confidence aligns with level 2 of Kirkpatrick's 4-level

Copyright © 2026 Huntsman et al. This is an open-access publication distributed under the terms of the Creative Commons Attribution license.

model of training evaluation.[14] Melanoma-related content was assessed through the weekly course quiz, alongside material taught outside of PBL that week, as well as in the end-of-block examination administered 4 weeks later. Specific question content is not reported here to preserve item integrity for future iterations. Future directions include incorporating additional test questions and broader assessment measures. Further limitations include a survey response rate of 46.6%, which may have introduced response bias, as students with more favorable views were more likely to participate. Women comprised 64.4% of respondents, a higher proportion than the student class (54%), and the majority of both respondents (72.9%) and the student body (74%) identified as White. This lack of racial and ethnic diversity limits the generalizability of our findings to broader medical student populations. While acknowledging these limitations, the overall feedback from this study was highly positive.

This PBL case, centered on a patient with a nonhealing foot ulcer ultimately diagnosed as acral melanoma, offered students a valuable opportunity to develop their clinical reasoning skills. It was well received by learners, challenged common assumptions about melanoma, and moved beyond the textbook by incorporating social determinants of health, histopathology, diagnostic imaging, and molecular oncology. The case is adaptable across institutions, and it is our hope that other medical schools will adopt this case within hybrid or fully integrated PBL curricula, further strengthening student preparation for the early recognition of melanoma.

Appendices

A. PBL Case Facilitator Guide.docx

B. Student Materials.docx

C. Student Survey.docx

All appendices are peer reviewed as integral parts of the Original Publication.

Annabelle Huntsman: Medical Student, Spencer Fox Eccles School of Medicine at the University of Utah

Amanda Jiang: Medical Student, Spencer Fox Eccles School of Medicine at the University of Utah

Justin Lyon: Medical Student, Spencer Fox Eccles School of Medicine at the University of Utah

Rachel R. Codden, MPH: Data Analyst, Division of Epidemiology, Department of Internal Medicine, Spencer Fox Eccles School of Medicine at the University of Utah

Morgan M. Millar, PhD: Associate Professor, Division of Epidemiology, Department of Internal Medicine, Spencer Fox Eccles School of Medicine at the University of Utah

Dekker C. Deacon, MD, PhD: Assistant Professor, Department of Dermatology, Spencer Fox Eccles School of Medicine at the University of Utah

Sarah D. Cipriano, MD, MPH, MS: Associate Professor, Department of Dermatology, Spencer Fox Eccles School of Medicine at the University of Utah; ORCID: https://orcid.org/0000-0003-0888-1458

Disclosures
None to report.

Funding/Support
None to report.

Ethical Approval
The University of Utah Institutional Review Board reviewed this project.

References

1. Trullàs JC, Blay C, Sarri E, Pujol R. Effectiveness of problem-based learning methodology in undergraduate medical education: a scoping review. *BMC Med Educ*. 2022;22(1):104. https://doi.org/10.1186/s12909-022-03154-8
2. Ge WL, Zhu XY, Lin JB, et al. Critical thinking and clinical skills by problem-based learning educational methods: an umbrella systematic review. *BMC Med Educ*. 2025;25(1):455. https://doi.org/10.1186/s12909-025-06951-z
3. Suter B, Riedel H. Problem-based learning: cervical adenocarcinoma three-session case for first-year medical students—patient Minnie Pauls. *MedEdPORTAL*. 2017;13:10561. https://doi.org/10.15766/mep_2374-8265.10561
4. Lawson L, Stair R. Mysterious cyanosis: a PBL case on methemoglobinemia. *MedEdPORTAL*. 2015;11:10042. https://doi.org/10.15766/mep_2374-8265.10042
5. Wade T, Webb T. Diagnosis and management of shock: a PBL exercise. *MedEdPORTAL*. 2012;8:9092. https://doi.org/10.15766/mep_2374-8265.9092
6. Clifton M. Adolescent pelvic inflammatory disease problem based learning (PBL). *MedEdPORTAL*. 2007;3:264. https://doi.org/10.15766/mep_2374-8265.264
7. Koestler J, Waite E, Chietero M, et al. Problem-based learning (PBL): abdominal pain in a pregnant woman. *MedEdPORTAL*. 2009;5:517. https://doi.org/10.15766/mep_2374-8265.517
8. Lim HW, Collins SAB, Resneck JS Jr, et al. The burden of skin disease in the United States. *J Am Acad Dermatol*. 2017;76(5): 958-972.e2. https://doi.org/10.1016/j.jaad.2016.12.043

Copyright © 2026 Huntsman et al. This is an open-access publication distributed under the terms of the Creative Commons Attribution license.

9. Mayer JE, Swetter SM, Fu T, Geller AC. Screening, early detection, education, and trends for melanoma: current status (2007-2013) and future directions: Part II. Screening, education, and future directions. *J Am Acad Dermatol*. 2014;71(4):611.e1-611.e10;quiz 621-2. https://doi.org/10.1016/j.jaad.2014.05.045
10. Cortez JL, Vasquez J, Wei ML. The impact of demographics, socioeconomics, and health care access on melanoma outcomes. *J Am Acad Dermatol*. 2021;84(6):1677-1683. https://doi.org/10.1016/j.jaad.2020.07.125
11. Mangion SE, Phan TA, Zagarella S, Cook D, Ganda K, Maibach HI. Medical school dermatology education: a scoping review. *Clin Exp Dermatol*. 2023;48(6):648-659. https://doi.org/10.1093/ced/llad052
12. Leeds FS, Atwa KM, Cook AM, Conway KA, Crawford TN. Teaching heuristics and mnemonics to improve generation of differential diagnoses. *Med Educ Online*. 2020;25(1):1742967. https://doi.org/10.1080/10872981.2020.1742967
13. Harris PA, Taylor R, Minor BL, et al. The REDCap consortium: building an international community of software platform partners. *J Biomed Inform*. 2019;95:103208. https://doi.org/10.1016/j.jbi.2019.103208
14. Kirkpatrick JD, Kirkpatrick WK. *Kirkpatrick's Four Levels of Training Evaluation*. Association for Talent Development; 2016.

Received: October 28, 2025
Accepted: April 15, 2026
Published: June 2, 2026

Copyright © 2026 Huntsman et al. This is an open-access publication distributed under the terms of the Creative Commons Attribution license.
